# Causal relationship between thyroid dysfunction and gastric cancer: a two-sample Mendelian randomization study

**DOI:** 10.3389/fendo.2024.1335149

**Published:** 2024-04-26

**Authors:** Qi Zhang, Yongliang Mu, Xin Jiang, Yirui Zhao, Qiutao Wang, Zhen Shen

**Affiliations:** ^1^ Department of Gastrointestinal and Colorectal Surgery, China-Japan Union Hospital of Jilin University, Changchun, Jilin, China; ^2^ Department of Thyroid Surgery, China-Japan Union Hospital of Jilin University, Changchun, Jilin, China

**Keywords:** hyperthyroidism, hypothyroidism, FT4, TSH, gastric cancer, Mendelian randomization, causal relationship

## Abstract

**Backgroud:**

Gastric cancer is one of the most common cancers worldwide, and its development is associated with a variety of factors. Previous observational studies have reported that thyroid dysfunction is associated with the development of gastric cancer. However, the exact relationship between the two is currently unclear. We used a two-sample Mendelian randomization (MR) study to reveal the causal relationship between thyroid dysfunction and gastric cancer for future clinical work.

**Materials and methods:**

This study is based on a two-sample Mendelian randomization design, and all data are from public GWAS databases. We selected hyperthyroidism, hypothyroidism, free thyroxine (FT4), and thyroid-stimulating hormone (TSH) as exposures, with gastric cancer as the outcome. We used three statistical methods, namely Inverse-variance weighted (IVW), MR-Egger, and weighted median, to assess the causal relationship between thyroid dysfunction and gastric cancer. The Cochran’s Q test was used to assess the heterogeneity among SNPs in the IVW analysis results, and MR-PRESSO was employed to identify and remove IVs with heterogeneity from the analysis results. MR-Egger is a weighted linear regression model, and the magnitude of its intercept can be used to assess the horizontal pleiotropy among IVs. Finally, the data were visualized through the leave-one-out sensitivity test to evaluate the influence of individual SNPs on the overall causal effect. Funnel plots were used to assess the symmetry of the selected SNPs, forest plots were used to evaluate the confidence and heterogeneity of the incidental estimates, and scatter plots were used to assess the exposure-outcome relationship. All results were expressed as odds ratios (OR) and 95% confidence intervals (95% CI). P<0.05 represents statistical significance.

**Results:**

According to IVW analysis, there was a causal relationship between hypothyroidism and gastric cancer, and hypothyroidism could reduce the risk of gastric cancer (OR=0.936 (95% CI:0.893-0.980), P=0.006).This means that having hypothyroidism is a protective factor against stomach cancer. This finding suggests that hypothyroidism may be associated with a reduced risk of gastric cancer.Meanwhile, there was no causal relationship between hyperthyroidism, FT4, and TSH and gastric cancer.

**Conclusions:**

In this study, we found a causal relationship between hypothyroidism and gastric cancer with the help of a two-sample Mendelian randomisation study, and hypothyroidism may be associated with a reduced risk of gastric cancer, however, the exact mechanism is still unclear. This finding provides a new idea for the study of the etiology and pathogenesis of gastric cancer, and our results need to be further confirmed by more basic experiments in the future.

## 1 Introduction

Gastric cancer is currently one of the most common cancers in the world and the second most common cause of cancer-related deaths (1). Its incidence is associated with region, race, gender, dietary habits, and genetic factors, etc. Some factors have been found to increase the risk of gastric cancer, such as familial genetic history, Helicobacter pylori infection, smoking, alcohol consumption, dietary habits, and EBV infection, etc. (2–5). In the recent years, researchers have found that thyroid-related hormones are associated with the risk of gastric cancer.Puhr et al. (6)found that elevated FT4 was associated with poorer overall survival in patients with advanced gastro-oesophageal cancer. Dore et al. (7) found that hypothyroidism was associated with an increased risk of gastric cancer in male patients. The above studies indicated an association between thyroid dysfunction and gastric cancer, but a causal association could not be deduced, it is necessary to explore the etiology and pathogenesis of the relationship between thyroid dysfunction and gastric cancer.

The advantage of the MR study is that it exploits the “randomness” of genetic variation, based on the Mendelian law of inheritance that homologous chromosomes are randomly assigned and freely combine during meiosis at the phase of zygote, and inferring the causal relationship between genotype and phenotype by observing the distribution of alleles on chromosomes in a population. It can well overcome the limitations of observational studies, such as confounding factors and reverse causality interference. MR study are more economical and convenient than randomised controlled trials(RCT), and indirectly explore the potential causal relationship between thyroid dysfunction and gastric cancer without the involvement of a trial, and provide evidence that is closer to that of randomised controlled trials than that of observational studies. However MR study has a series of drawbacks: On the one hand, it does not offer the same degree of generalizability and robustness as RCT, it can only approximate RCT rather than replace them completely. On the other hand, it has a limited application, being used only in situations with high genetic variance explained and large sample sizes, and for risk factors that are strongly influenced by environmental factors, MR studies may not be sufficient to accurately assess the influence of genetic variables on them.

In recent years, genome-wide association studies (GWAS) have been widely used to study the relationship between human genetic variation and disease risk. In two-sample MR studies, analyzing GWAS data from different study populations and selecting single-nucleotide polymorphisms closely associated with phenotypes as instrumental variables. This approach avoids the limitation of single-sample MR studies where data come from a single source, making the results vulnerable to confounding factors. Additionally, the larger sample size enhances the study’s efficiency and statistical power, leading to more reliable and stable results.In this study, we chose hyperthyroidism, hypothyroidism, free thyroxine (FT4) and thyroid-stimulating hormone (TSH) as the exposure factors associated with thyroid dysfunction (FT4 and TSH are blood concentrations), and gastric cancer as the endpoint, and explored the potential causal relationship between thyroid dysfunction and gastric cancer by using a two-sample Mendelian randomisation analysis, to further study the pathogenesis of gastric cancer and to provide new ideas for future clinical treatment. provide new ideas for future clinical treatment.

## 2 Materials and methods

### 2.1 Study design and experimental data sources

The aim of this study was to investigate the causal relationship between thyroid dysfunction and gastric cancer. All data were obtained from publicly available GWAS databases, and ethical approval and informed consent were obtained for data from these studies, so there was no further need to obtain additional ethical approval and informed consent authorisation for this paper. This study was written according to the Report of Observational Studies in Epidemiology-MR (STROBE-MR) developed specifically for two-sample Mendelian randomisation studies (8).

In this study,we selected hyperthyroidism, hypothyroidism, free Thyroxine (FT4) and Thyrotropin (TSH) as the exposure. Hyperthyroidism (GWAS ID:ebi-a-GCST90018860, including 460499 samples and 24189279 SNPs in 3557 cases and 456942 controls) and hypothyroidism (GWAS ID:ebi-a-GCST90018862, including 410,141 samples and 24138872 SNPs in 30155 cases and 379986 controls) were obtained from the IEU Open GWAS Project database (https://gwas.mrcieu.ac.uk/). FT4 and TSH are blood concentrations, and their GWAS data are from the Thyroidomics Consortium database (https://transfer.sysepi.medizin.uni-greifswald.de/thyroidomics/datasets/), containing 72,167 samples.The GWAS data for gastric cancer as outcome were also obtained from the IEU Open GWAS Project database (GWAS ID:ebi-a-GCST90018849, including a total of 476,116 samples and 241,88662 SNPs in 1029 cases and 475087 controls). All subjects in the GWAS data were from European populations to avoid bias caused by ethnicity and regional and other relevant confounding factors causing bias.

### 2.2 Selection of instrumental variables

**Figure 1:**
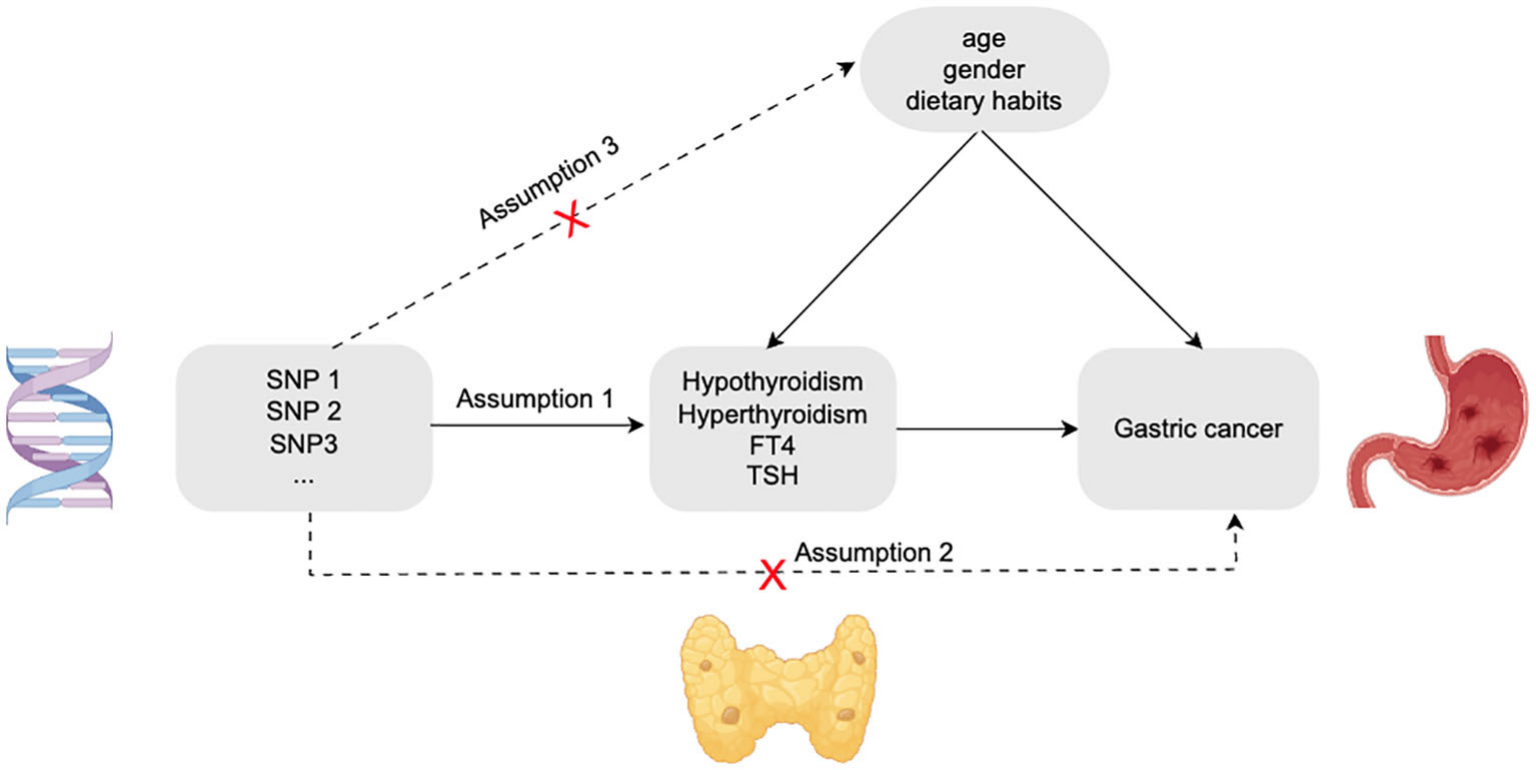
Three assumptions of the two-sample Mendelian Randomisation study.

We selected valid SNPs as instrumental variables for this study based on three assumptions (**Figure 1**). First. Each SNP was significantly correlated with the phenotype of thyroid dysfunction and could represent this type of disease well; Second. Each SNP associated with thyroid dysfunction does not have a direct relationship with gastric cancer and will not have any effect on the risk of gastric cancer; Third. These SNPs were also not associated with any potential confounding factors (e.g.,age, gender, dietary habits, etc.) (9).

Based on the above hypothetical conditions, we obtained GWAS data related to thyroid dysfunction and gastric cancer from databases, then selected SNPs for the GWAS data of thyroid dysfunction (hyperthyroidism, hypothyroidism, FT4, and TSH). To ensure significant correlation between SNP and thyroid dysfunction, all SNPs included in this study satisfied P<5*10-8. In the study, to avoid the potential bias due to linkage disequilibrium (LD) among SNPs, we adopted a reference threshold of LD r2<0.001 and set the distance between each SNP to 10000 kb to ensure their mutual independence (10). Secondly, to eliminate, the interference of potential horizontal pleiotropy of each SNP on the outcome, we used the GWAS catalogue in the EBI database (https://www.ebi.ac.uk/gwas/) as well as the PhenoScanner database (http://www.phenoscanner.medschl.cam.ac.uk/) to retrieve the phenotype of each SNP included above and remove SNPs associated with gastric cancer phenotypes to ensure that instrumental variables were not confounded by confounders (11). The acquired SNPs for thyroid dysfunction and gastric cancer were coordinated to remove palindromes and incompatible alleles (12). Finally, weak instrumental variables were excluded. By calculating the F statistic value of each SNP, if the F statistic value of SNPs is<10, it means that the SNP is a weak instrumental variable, which should be eliminated to improve the accuracy and credibility of the study results and to ensure the validity of causal inference (13, 14). The formula for calculating the F statistic value of each SNP is as follows (14):


$$F=(\frac{R^{2}}{1-R^{2}})(\frac{N-K-1}{K})$$

R is calculated as follows:


$${\text{R}}^{2}=2\times \left(1-\text{MAF}\right)\times \text{MAF}\times {\left(\text{β}\right)}^{2}$$

N: GWAS data samples;

K: the number of SNPs;

R^2^: Cumulative explained variance of the selected instrumental variables at exposure;

MAF: The frequency of the minor alleles of the selected SNPs;

β: Effect values of alleles;

### 2.3 Statistical analysis

The analysis of all MR data in this study was based on the Two Sample MR package (version 0.5.7) in R software (version 4.3.1). We chose Inverse-variance weighted (IVW), MR-Egger, and weighted median as the three statistical methods to assess the causal effect between thyroid dysfunction and gastric cancer. IVW allows for the weighted aggregation of Wald estimates for each SNP in the statistical data, which makes the results more precise and stable, with the most reliable statistical validity (15, 16). Therefore, we chose inverse-variance weighted as the primary method for assessing the causal relationship between thyroid dysfunction and gastric cancer risk. mR-Egger and weighted median were used as additional complementary research methods (17).

**Figure 2:**
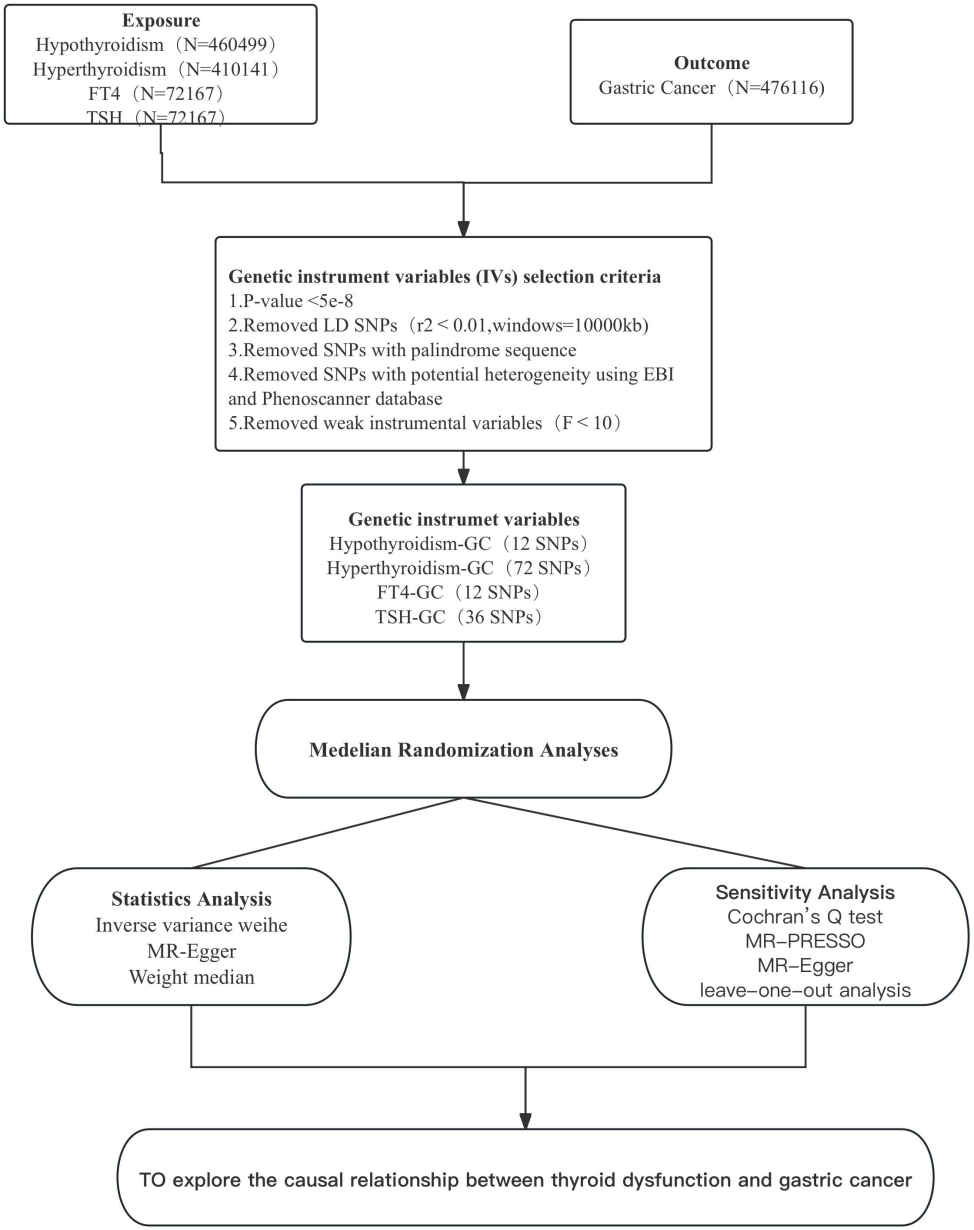
Flowchart of this two-sample Mendelian randomisation study.

Considering the possible heterogeneity among instrumental variables, we chose Cochran’s Q test to assess whether there was heterogeneity among SNPs in the results of IVW analysis. If P<0.05, it indicates the presence of instrumental variables with heterogeneity in the results (18). We used MR-PRESSO to identify and remove IVs with heterogeneity from the analysis results. the remaining IVs were then analysed again by the IVW method to obtain the final results. mR-Egger is a weighted linear regression model, and the magnitude of its intercept can be used to assess whether there is horizontal pleiotropy between IVs, where an intercept value close to 0 and a P value > 0.05 indicates no horizontal pleiotropy (19). Finally, the data were graphically visualised to assess the influence of individual SNPs on the overall causal effect by the leave-one-out analysis (20), with funnel plots to assess the symmetry of the selected SNPs, forest plots to assess the confidence and heterogeneity of the chance estimates, and scatter plots to assess the relationship between the effect of exposure and outcome. All results were expressed as odds ratio (OR) and 95% confidence interval (95% CI). p<0.05 represents statistical significance. **Figure 2** shows the MR flow chart of this study as a whole.

## 3 Results

### 3.1 Selection of instrumental variables

After excluding linkage disequilibrium, F-value screening and adjusting for allelic conditions, 12 SNPs associated with hyperthyroidism and gastric cancer, 72 SNPs associated with hypothyroidism and gastric cancer, 12 SNPs associated with FT4 and gastric cancer, and 36 SNPs associated with TSH and gastric cancer were obtained, and these were selected as instrumental variables for this study.

### 3.2 MR analysis of thyroid dysfunction and gastric cancer

We assessed whether there is a causal relationship between hyperthyroidism, hypothyroidism, FT4 and TSH and the risk of gastric cancer by using IVW, MR-Egger and Weight median studies. Based on IVW results, this study suggests a causal relationship between hypothyroidism and gastric cancer. Hypothyroidism can reduce the risk of developing gastric cancer (OR=0.936 (95% CI:0.893-0.980); P=0.006<0.05), while MR Egger and Weighted Median did not suggest statistical significance. Also, when assessing the causal relationship between hyperthyroidism, FT4 and TSH and the risk of gastric cancer, none of the three methods suggested statistical significance (**Table 1**)

**Table 1:** MR estimates by different methods for assessing the causal effect of thyroid dysfunction on Gastric Cancer.

Exposure	MR methods	nSNP	Beta	se	OR (95%CI)	P-value
Hyperthyroidism	MR Egger	12	0.114	0.185	1.121 (0.780,1.610)	0.5501
	Weighted median	12	-0.071	0.043	0.931 (0.854,1.014)	0.098
	IVW	12	-0.025	0.057	0.976 (0.872,1.092)	0.669
Hypothyroidism	MR Egger	72	-0.016	0.055	0.984 (0.884,1.095)	0.766
	Weighted median	72	-0.061	0.041	0.941 (0.868.1.019)	0.136
	IVW	72	-0.066	0.024	0.936 (0.893,0.980)	0.006
FT4	MR Egger	12	0.295	0.165	1.343 (0.972,1.856)	0.395
	Weighted median	12	0.203	0.963	1.225 (1.017,1.474)	0.035
	IVW	12	0.157	0.076	1.170 (1.008,1.358)	0.104
TSH	MR Egger	36	0.153	0.187	1.165 (0.807.1.681)	0.421
	Weighted median	36	0.073	0.077	1.075 (0.925,1.250)	0.347
	IVW	36	0.099	0.073	1.105 (0.957,1.275)	0.175

### 3.3 Heterogeneity, pleiotropy, and sensitivity analysis

**Figure 3:**
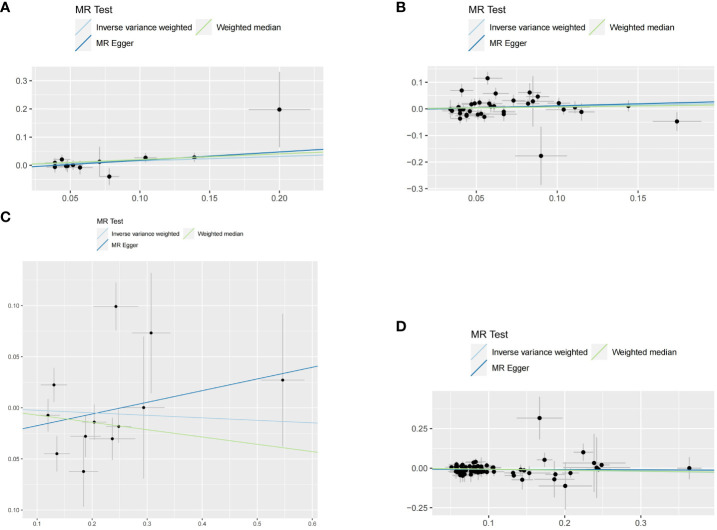
Scatter plots for this MR analyses of the causal relationship between thyroid dysfunction and gastric cancer: **(A)** FT4-GC;**(B)** TSH-GC;**(C)** Hyperthyroidism-GC;**(D)** Hypothyroidism-GC.

**Figure 4:**
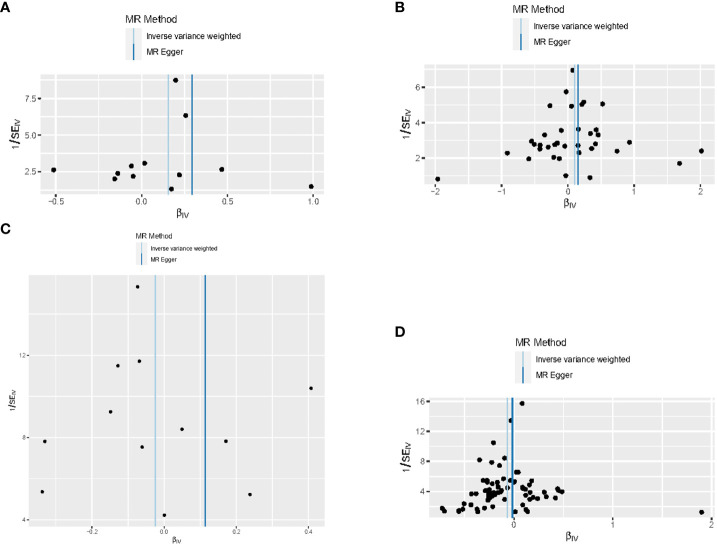
Funnel plots for this MR analyses of the causal relationship between thyroid dysfunction and gastric cancer: **(A)** FT4-GC;**(B)** TSH-GC;**(C)** Hyperthyroidism-GC;**(D)** Hypothyroidism-GC.

**Figure 5:**
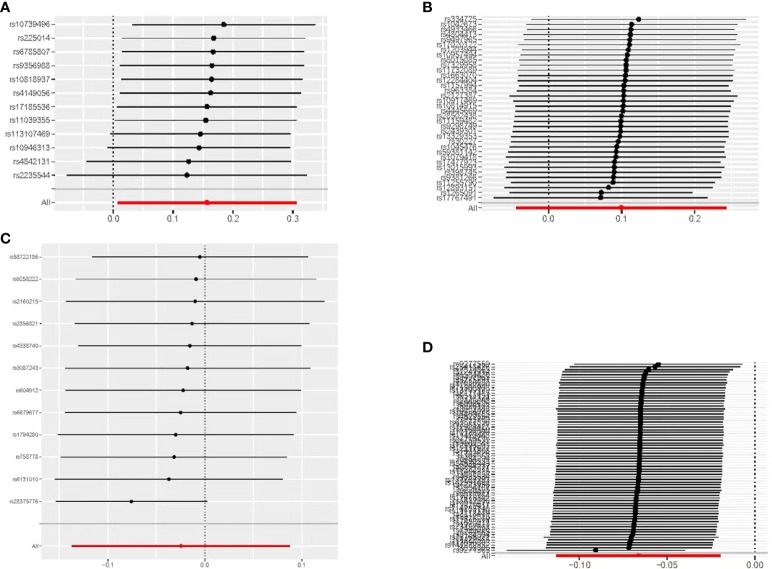
MR leave-one-out sensitivity analyses of the causal relationship between thyroid dysfunction and gastric cancer: **(A)** FT4-GC;**(B)** TSH-GC;**(C)** Hyperthyroidism-GC;**(D)** Hypothyroidism-GC.

When hypothyroidism and FT4 were used as exposures, the results of the IVW method were evaluated by Cochran’s Q test, which showed P > 0.05, indicating that there was no heterogeneity among the SNPs in the results (**Table 2**), which confirmed the reliability of the results of the MR analysis. In contrast, when hyperthyroidism and TSH were used as exposures, the results of Cochran’s Q test showed P< 0.05, which indicated that there was heterogeneity among the SNPs in the presence of hyperthyroidism and TSH, and that these heterogeneities could be caused by a variety of factors, including differences in the distribution of genotypes between populations from which the data of the two-sample study originated, due to the presence of genetic heterogeneity. Secondly, heterogeneity may also be caused by differences in environmental factors and lifestyles among different populations, while differences in sample size can also lead to heterogeneity. When heterogeneity existed, the random effects model in IVW was chosen for data analysis (18). MR-Egger regression analysis showed that hyperthyroidism, hypothyroidism, TSH and FT4 all showed P > 0.05, indicating that all SNPs included in the study were not affected by polytropy at the genetic level (**Figure 3**). The funnel plot showed that the scatter points representing each SNPs were basically symmetrical, and there was no potential bias in the results (**Figure 4**); the results of the leave-one-out test showed that after excluding each SNP sequentially, the results of IVW analyses of the remaining SNPs were similar to the results of analyses with the inclusion of all the SNPs, and no SNPs were found to have a large impact on the assessment of causal associations, which further confirmed the stability of the results (**Figure 5**). Therefore, we consider the results of the IVW analyses to be reliable.

**Table 2:** Sensitivity analysis of the causal relationship between thyroid dysfunction and Gastric Cancer.

Exposure	Pleiotropy		Heterogeneity	
	Horizontal pleiotropy (Egger intercept)	Horizontal pleiotropy (P)	Heterogeneity (Q)	Heterogeneity (P-value)
Hyperthyroidism	-0.029	0.448	36.961	1.17E-04
Hypothyroidism	-0.006	0.314	70.620	0.490
FT4	-0.012	0.366	7.612	0.748
TSH	-0.004	0.759	77.155	5.219E-05

## 4 Discussion

In our two-sample MR study, a causal relationship between hypothyroidism and gastric cancer was revealed. This shows that having hypothyroidism is a protective factor against gastric cancer, a finding that suggests hypothyroidism may be associated with a reduced risk of gastric cancer.

Several studies shared the same results as the present study. A prospective study on mild hypothyroidism induced by propylthiouracil found that hypothyroidism was associated with regression of the primary brain tumour and a significantly prolonged survival time (21). Puhr et al. (22) found that patients treated with thyroid hormone replacement therapy had a longer overall survival than those who did not receive thyroid hormone replacement therapy (51.3 vs. 30.6 months), which suggests that hypothyroidism and its replacement therapy may be a potential factor influencing the prognosis of patients.Hercbergs et al. (23) found that spontaneous hypothyroidism delayed the onset of cancer or reduced its aggressiveness. Thyroid hormone analogues have also shown potent thyrotropic activity, e.g., triiodothyronine acetate, an acetate metabolite of T3, and tetraiodothyronine acetate, a derivative of T4, reduce the risk of cancer progression, enhance the efficacy of therapy, and inhibit cancer recurrence (24).

Thyroid hormones play an important role in the regulation of growth, development and metabolism of the body. Thyroid hormones and thyroid hormone receptors have been found to play a role in promoting cell proliferation and pro-angiogenesis through integrin αvβ3 mediated by phosphatidylinositol-3-kinase and MAPK (25).Kathrin et al. (26) compared the effects of thyroid hormones in two mouse xenograft tumour models positive and negative for integrin αvβ3 expression. Integrin αvβ3-positive human interstitial thyroid cancer cells SW1736 and integrin αvβ3-negative human hepatocellular carcinoma cells HuH7 were injected into the flanks of nude mice. It was found that mice with hypothyroidism and treated with Trac had reduced angiogenesis, significantly slower tumour growth and therefore prolonged survival compared to mice with normal thyroid function. Circulating levels of Free T4 in humans are higher than those of T3, and the hormone receptor affinity of T4 for αvβ3 is also superior to that of T3. it has been found that the T4 playing a major role in stimulating tumour cell proliferation as well as angiogenic process plays a major role (27). Thyroid hormone status can stimulate cancer cell proliferation and invasion, and thyroid hormones have been found to stimulate cancer cell growth and metastasis in rodent research models, which is inhibited by hypothyroidism (28). TRs have been reported to be tumour suppressive, with 96% of nuclear TRβ1 expression detected in normal epithelial cells, but significantly less frequent in adenomas (~83%) and malignant tumours (~68%) than in normal tissues (29). Also loss of normal thyroid receptor function regulates tumour development and progression. This is not only the case in gastric cancer, but the available epidemiology suggests that reduced levels of thyrotropic hormone can lead to an increased risk of lung, breast, colon, prostate, and breast cancers (30, 31). In a two-sample Mendelian randomisation study of thyroid dysfunction and breast cancer conducted by Yuan et al. (32), it was found that hypothyroidism also reduces breast cancer risk of the disease. The presence of hypothyroidism has also been noted to improve clinical response to treatment and reduce tumour growth in studies of patients with melanoma, renal cell carcinoma, glial carcinoma and inert breast cancer (33).

Interestingly, a cross-sectional study based on a database of the Spanish population (Base de Datos Clínicos de Atención Primaria, BDCAP) found that the relative risk of cancer was higher in patients diagnosed with hypothyroidism than in the normal population, and that the risk was higher in men than in women. However, it was also found that in people aged 65 years and older, hypothyroid patients had a reduced risk of gastric, bladder, and colorectal cancers (34). This is not exactly the same as in the present study, and the possible reasons for this are differences in sample size and problems associated with selection bias.

All the samples in our two-sample Mendelian randomisation study were from European populations, which avoided the bias caused by ethnic and regional differences, and also reduced the interference of confounding factors and the reverse causality between exposure and outcome, and the results of the study have a certain degree of reliability, and can provide a reference for more basic research in the future. There are some shortcomings in our study, for example, the incidence of gastric cancer is also affected by region and race, and whether the causal relationship between hypothyroidism and gastric cancer among different regions and races is consistent with the results of the present study needs to be further explored in more studies in the future.

## 5 Conclusion

With the help of a two-sample Mendelian randomisation study, we found that there is a causal relationship between hypothyroidism and gastric cancer, and that hypothyroidism may be associated with a reduced risk of gastric cancer; however, the exact mechanism is still unclear. This finding provides new ideas for the study of the etiology and pathogenesis of gastric cancer.Our findings need to be further confirmed by more basic experiments in the future.

## Data availability statement

The original contributions presented in the study are included in the article/supplementary material. Further inquiries can be directed to the corresponding author.

## Author contributions

QZ: Writing – original draft, Writing – review & editing. YM: Writing – original draft. XJ: Data curation, Writing – original draft. YZ: Visualization, Writing – original draft. QW: Data curation, Writing – original draft. ZS: Writing – review & editing.
